# PROFESSIONAL CAREGIVERS’ PERCEPTIONS OF PEOPLE WITH DEMENTIA AT ADULT DAY SERVICES IN TAIWAN: THE EFFECTS ON CLIENTS

**DOI:** 10.1093/geroni/igz038.1377

**Published:** 2019-11-08

**Authors:** Chih-ling Liou

**Affiliations:** Kent State University, North Canton, Ohio, United States

## Abstract

This study aims to explore staff perception of their clients with dementia in five ADS centers in Taiwan. Focused ethnography was used to collect data from 45 individual interviews with nurse’s aides, nurses, social workers, housekeepers, volunteers, bus drivers and centers’ directors and 600 hours of field observations. The findings from the content analysis revealed two themes reflecting ADS staff’s attitudes towards clients: (1) labeling the clients as “old” or “sick” to distance themselves from the clients and (2) viewing the clients as their aging relatives and over-helping them. Both attitudes not only affected staff-client relationships but also influenced how clients viewed themselves by inducing self-labeling of incompetent and dependent. More dementia-specific and communication training is needed for staff at ADS centers in Taiwan to change their attitudes and behavior toward clients with dementia. With a more positive attitude of people with dementia, the prerequisites for person-centered care will improve.

